# Temporal Dynamics of Cerebral Blood Flow During the Acute Course of Severe Subarachnoid Hemorrhage Studied by Bedside Xenon-Enhanced CT

**DOI:** 10.1007/s12028-019-00675-x

**Published:** 2019-02-21

**Authors:** Henrik Engquist, Elham Rostami, Per Enblad

**Affiliations:** 1 Department of Neuroscience/Neurosurgery, Uppsala University, Uppsala, Sweden; 2 Department of Surgical Sciences/Anesthesia and Intensive Care, Uppsala University Hospital, 751 85 Uppsala, Sweden

**Keywords:** Subarachnoid hemorrhage, Delayed cerebral ischemia, Cerebral blood flow, Xenon–CT, XeCT, Temporal, Sequential

## Abstract

**Background:**

Compromised cerebral blood flow (CBF) is a crucial factor in delayed cerebral ischemia after subarachnoid hemorrhage (SAH). Repeated measurement of CBF may improve our understanding of the temporal dynamics following SAH. The aim of this study was to assess CBF at different phases of the acute course in poor-grade SAH patients, hypothesizing more pronounced disturbances at day 4–7, and that the initial level of CBF determines the following course of CBF.

**Methods:**

Mechanically ventilated SAH patients were scheduled for bedside measurement of regional and global cortical CBF at day 0–3, 4–7, and 8–12, using xenon-enhanced computed tomography in a mobile setup. Patients were dichotomized depending on high or low initial global cortical CBF and cutoff level 30 ml/100 g/min.

**Results:**

Eighty-one patients were included, and 51 had measurements at day 0–3 and 4–7. In patients with high initial CBF, the level was unchanged at day 4–7; 37.7 (IQR 32.6–46.7) ml/100 g/min versus 36.8 (IQR 29.5–44.8). The low-CBF group showed a slight increase from 23.6 (IQR 21.0–28.1) ml/100 g/min to 28.4 (IQR 22.7–38.3) (*P* = 0.025), still markedly lower than the high-CBF group (*P* = 0.016). In the low-CBF group, CBF increased in patients who received hypertension, hypervolemia, and hemodilution (HHH therapy) but remained low in standard treated patients. For the subset of 27 patients examined also at day 8–12, the differences depending on initial CBF level were no longer statistically significant. Among patients with still low CBF at day 4–7, the proportion who had poor short-term outcome was 55% compared to 35% (n.s.) for patients with high CBF.

**Conclusions:**

CBF studied in poor-grade SAH patients *at large* did not show any statistically significant changes over time. Stratifying patients by high or low initial CBF and whether HHH therapy was given revealed an association between low initial CBF and persistent low CBF at day 4–7. These findings may be of clinical relevance in managing SAH patients with low early CBF.

## Introduction

Aneurysmal subarachnoid hemorrhage (SAH) carries a significant risk of both severe neurological sequelae and mortality despite improved techniques for early aneurysm repair and modern neurosurgical intensive care [1, 2]. There is still insufficient understanding of the pathophysiological mechanisms triggered already at the onset of the hemorrhage, leading to a various degree of secondary brain injury and development of delayed cerebral ischemia (DCI) [3]. The classic explanation for disturbed cerebral blood flow (CBF) has been vasospasm caused by degradation products from blood extravasated in the subarachnoid space [4]. Even if a more complex explanatory model has evolved, including inflammatory processes, endothelial dysfunction, and microvascular disturbances [5], compromised CBF is a crucial factor in the development of DCI [6, 7]. Therefore, repeated measurement of global and regional CBF may contribute to the understanding of the temporal dynamics of CBF in the acute course of the disease and potentially help in the clinical management of these patients.

The aim of the present study was to prospectively assess global and regional CBF at different time phases during the acute course of SAH in a cohort of poor-grade, mechanically ventilated patients. The primary hypothesis was that CBF disturbances are more pronounced at day 4–7 compared to day 0–3 and that there is a gradual restitution of CBF at day 8–12. A second hypothesis was that the initial level of CBF determines the following temporal course of CBF. The influence on CBF from therapeutic hypertension, hypervolemia, and hemodilution (HHH therapy) given to patients with clinical suspicion of DCI was also addressed.

## Materials and Methods

### Patients and Study Protocol

Unconscious patients with SAH, treated in our neuro-intensive care (NIC) unit, are as a routine scheduled for measurement of CBF using bedside xenon-enhanced computed tomography (XeCT) at day 0–3, 4–7, and 8–12. As mechanical ventilation is mandatory for the bedside XeCT procedures in our setting to ensure patient safety and image quality, only unconscious patients with severe SAH requiring intubation are examined. Patients who improve sufficiently to be extubated in the early course have no further CBF measurements.

During the time period 2013–2016, patients with CT verified spontaneous SAH and a valid baseline XeCT CBF measurement within day 0–3 were prospectively included in this study, and clinical data and measurements were collected accordingly. Patients with further XeCT procedures also at day 4–7 or at day 4–7 *and* 8–12 were allocated into subgroups (*subgroup 04* and *048*, respectively) to enable comparison of CBF over time. Exclusion criteria were severe intracranial hypertension, deep sedation with thiopental, respiratory problems requiring FiO2 > 0.6 (which precludes XeCT), futility or “do not resuscitate” order, and inability to obtain consent from the patients next of kin.

### Clinical Management

The standardized NIC protocol for management of SAH in our unit was applied for all patients, including continuous monitoring of physiological and biochemical parameters and clinical surveillance for early identification of avoidable factors with prompt interventions to minimize secondary brain injury [8]. All patients included were intubated due to their neurological state at admission or following deterioration day 0–1. Sedation with propofol (Propolipid^®^, Fresenius Kabi AB, Uppsala, Sweden) was titrated in the interval 0–4 mg/kg/h, and morphine (Morfin, Meda AB, Solna, Sweden) administered as needed. As all patients had altered level of consciousness, they all received a ventriculostomy catheter to enable monitoring of intracranial pressure (ICP) and drainage of CSF. Elevated ICP (> 20 mmHg) was treated with open ventricular drainage against a pressure level of 15 mmHg. Aneurysms were treated early, preferably with endovascular coil embolization if feasible, or with surgical clipping.

After securing of the aneurysm, patients were kept mildly hypervolemic if not contraindicated by intracranial mass effect or elevated ICP. The volume status was maintained by fluid administration in the higher normal range with the addition of albumin infusion (Flexbumin^®^ 200 mg/ml, Baxter AG, Vienna, Austria) if needed and monitored by clinical evaluation, central venous pressure, and attentive fluid balance calculation. The mean arterial pressure (MAP) was kept above 85 mmHg using dobutamine (Hospira, Lake Forest, IL, USA) as inotrope or, if insufficient, norepinephrine (Noradrenalin, Hospira Nordic AB, Stockholm, Sweden) as vasopressor. Nimodipine (Nimotop^®^, Bayer Pharma AG, Berlin, Germany) was administered from admission and onward as iv infusion 2 mg/h and later orally 60 mg every 4 h if the patient improved. Repeated wake-up tests were performed to monitor patients for general or focal neurological changes [9]. Transcranial Doppler measurement was not part of the clinical routine.

### DCI and HHH Therapy

In the case of neurological deterioration (new focal deficits or reduced level of consciousness), clinical diagnose of DCI was concluded if other causes were ruled out, and HHH therapy was initiated for 5 days. The HHH therapy in our NIC protocol is cautious with a moderately elevated blood pressure target while keeping the patient in supine position, and with further attention on ensuring adequate intravascular volume status under careful re-evaluation of side effects. In addition to standard therapy, daily infusions of 500 ml dextran solution (Rheomacrodex^®^, Meda AB, Solna, Sweden) and 200 ml albumin 200 mg/ml were administered if tolerated, targeting CVP 8–12 mmHg. Vasoactive agents were used if needed to maintain a systolic blood pressure above 140 mmHg (preferably dobutamine or, as second-line, norepinephrine).

### Xenon–CT CBF Measurements

Following our NIC protocol, all patients with confirmed diagnose of SAH and mechanical ventilation should undergo XeCT for mapping of CBF at day 0–3, 4–7, and 8–12 if logistically possible. As an advantage over several other methods, XeCT allows repeated high-resolution measurements of regional CBF at the bedside in the NIC unit [10]. The XeCT procedures are performed following the principles described by Gur et al. and Yonas et al. [11–14]. Inhaled xenon gas is rapidly dissolved in blood and tissues and acts as an inert contrast agent during the CT scans. The subsequent calculation of CBF is based on Kety’s [15, 16] application of the Fick principle; blood flow is proportional to inert gas uptake in tissue. The Enhancer 3000 xenon delivery system (Diversified Diagnostic Products Inc, Huston, USA) is connected to the ventilator and breathing circuit. Ventilator settings are adjusted to compensate for increased compressible volume and titrated to normal PaCO2. Non-radioactive Xe^131^ at a concentration of 28% is administered to the breathing circuit for about 4 ½ min, and CT scans synchronized to the xenon inhalation are obtained by a bedside CereTom scanner (Neurologica, Boston, USA). Local CBF in each pixel of the CT image is calculated (ml/100 g/min) and plotted as color-coded maps at four different levels of the brain, with eight scans per level—two at baseline and six enhanced during xenon wash-in. Levels with extensive artifacts from bone structures or coils/clips were excluded from calculation. Typically, three scan levels could be used for further calculations in each patient.

#### Calculation of XeCT Parameters

**Fig. 1:**
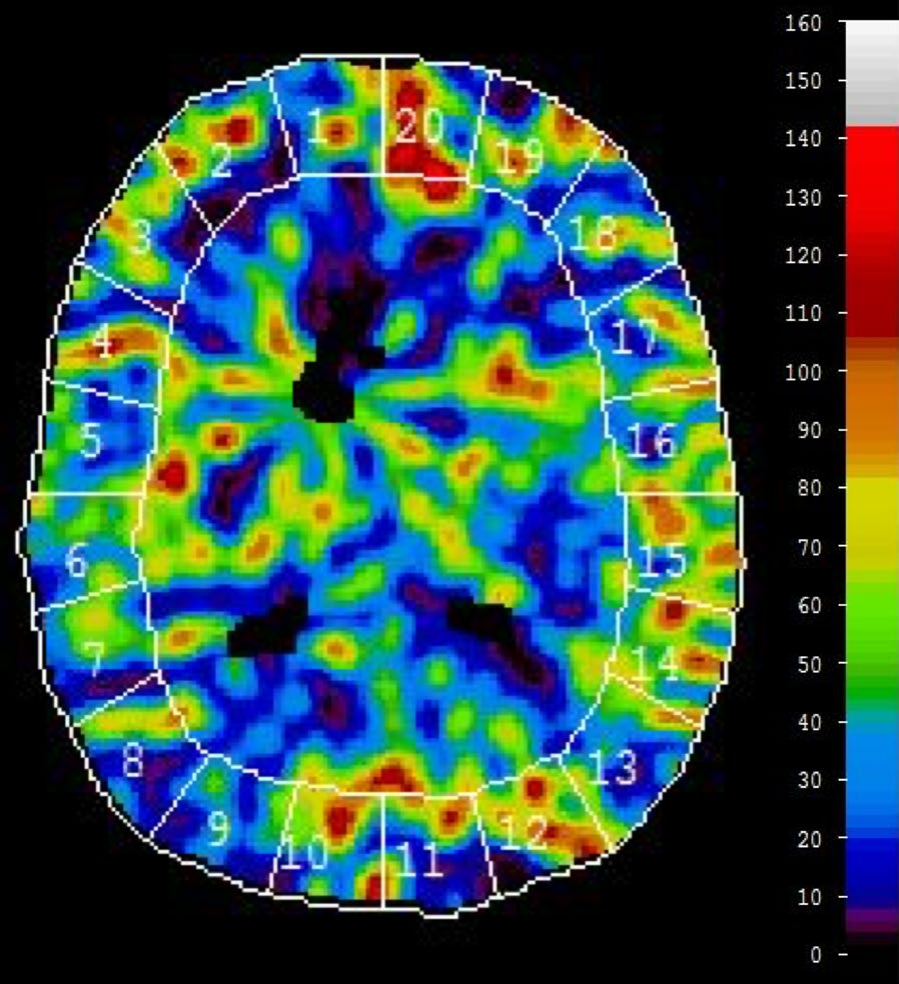
Regional CBF is determined for each of 20 cortical ROIs at three levels of the brain, resulting in 60 ROIs for further calculations. *Global cortical CBF* (ml/100 g/min) is calculated as mean of all valid ROIs at all scan levels (weighted average by ROI size). Example of CBF map 7 days after SAH from an aneurysm of the left posterior cerebral artery. The patient is in Hunt and Hess grade IV and is receiving HHH therapy at the time of measurement. Global CBF is 46.4 ml/100 g/min, and the proportion of low-flow ROI area (< 20 ml/100 g/min) is 1.3%

For the evaluation of CBF, a standardized set of XeCT CBF parameters were calculated for each patient [17]. Mean blood flow in each of twenty evenly distributed cortical regions of interest (ROIs) was calculated for every level (Fig. 1) [13], resulting in a total of sixty ROIs. Typical area of each ROI was 350–450 mm^2^. ROIs in areas of hematoma or with artifacts from ventricular drains were excluded from calculations.

*Global cortical CBF* (ml/100 g/min) was calculated as mean of all ROIs (weighted average by the individual ROI size) at all scan levels.

*CBF of the worst vascular territory* (rCBF worst) (ml/100 g/min) in each patient was identified and calculated as mean of the ROIs corresponding to the territory at all scan levels. The vascular territories were approximated as ROI 1–2 right anterior cerebral artery, ROI 3–8 right middle cerebral artery, ROI 9–10 right posterior cerebral artery and similarly for the left side territories (Fig. 1).

*The proportion of cortical ROI area with local blood flow below specified thresholds* was calculated to quantify the extent of regional area with low or near ischemic blood flow. These thresholds were set to 20 and 10 ml/100 g/min [18, 19], and the proportion of low-flow ROI area was calculated as sum of ROI area (mm^2^) with local CBF below the respective thresholds divided by the total analyzed ROI area in each patient.

To enable comparison over time in relation to the initial level of CBF, patients were *stratified depending on high or low early CBF* from the baseline XeCT measurements at day 0–3. The cutoff level was set at 30 ml/100 g/min for global cortical CBF, which is in the midrange of “oligemia” according to the previous literature [18], and initial CBF below this level has also been associated with worse outcome after SAH [20].

### Clinical Short-Term Outcome

To reflect short-term *clinical course outcome*, the neurological state at discharge from the NIC unit was determined as good (responding to commands, Glasgow Coma Score [GCS] motor 6), poor (unconscious, GCS motor ≤ 5), or dead. Follow-up native CT scans past day 12 (or latest available) were evaluated for infarcts and categorized according to the size of detected infarcts.

### Statistical Methods

SPSS statistics 23.0 software (IBM Corp, Armonk, NY, USA) was used for statistical analyses of the collected data. CBF data for groups of patients are presented as median values with interquartile range because of non-normal distribution. Statistical differences in CBF parameters between unrelated groups were tested with Mann–Whitney *U* test. In the analysis comparing measurements for patients with paired data from *two different* time windows, Wilcoxon signed ranks test was used (related samples). Friedman’s test was used to analyze data comparing measurements from *multiple* time windows. The Chi-squared test or Fisher’s exact test was used to compare proportions between groups. Statistical significance level was set at *P* < 0.05, and only *p* values below this level are displayed in text and figures.

## Results

### Patients Studied

During the study period, a total of 136 SAH patients underwent one or several XeCT procedures at different time windows in our NIC unit and were screened for inclusion. Eighty-one patients had valid *baseline* XeCT measurements at day 0–3 and were finally eligible for inclusion in the study. Fifty-one of these patients also had XeCT CBF measurements at day 4–7 (*subgroup 04*), and among those a further subgroup consisting of 27 patients had sequential measurements at both days 4–7 and 8–12 (*subgroup 048*). The clinical characteristics of the included patients are presented in Table 1. Mean age was 60 years (range 28–84) and 72% of the patients were female. Concerning the neurological state, 48% of the patients were in Hunt and Hess grade IV–V at admission, and as a number of patients deteriorated during day 0–1, 64% were in Hunt and Hess grade IV–V when graded at the time of the first XeCT. It was noted that the *subgroup 048* had the highest proportion of patients in Fisher grade 4 (78%) and in Hunt and Hess grade IV-V (74%) at the time of the baseline XeCT procedure.

**Table 1:** Characteristics for all included patients and for the subgroups of patients who had a second and third XeCT procedure at day 4–7 and 8–12; *subgroups 04 and 048*

Characteristic	All patients	Subgroup 04	Subgroup 048
	*n* (%)	*n* (%)	*n* (%)
Patients			
* n*	81	51	27
Gender			
* F*	58 (72)	35 (69)	16 (59)
Age (years)			
Mean (range)	60 (28–84)	60 (28–84)	59 (41–75)
Treatment modality			
Endovascular	65 (80)	39 (76)	22 (82)
Surgical clip	12 (15)	9 (18)	3 (11)
None	4 (5)	3 (6)	2 (7)
CT Fisher group			
3	23 (28)	14 (27)	6 (22)
4	58 (72)	37 (73)	21 (78)
Hunt and Hess grade at admission			
I–III	42 (52)	27 (53)	12 (44)
IV–V	39 (48)	24 (47)	15 (56)
Hunt and Hess at baseline XeCT			
III	29 (36)	16 (31)	7 (26)
IV–V	52 (64)	35 (69)	20 (74)
Hunt and Hess at XeCT day 4–7			
III		15 (29)	5 (18)
IV–V		36 (71)	22 (82)
Clinical diagnosis of DCI	26 (32)	22 (43)	15 (56)

The number of patients and percentage for each group and characteristic are presented; Fisher group—amount of blood at first CT, Hunt and Hess grade—neurological state at admission, at the time of inclusion (at baseline XeCT day 0–3), and at XeCT day 4–7

*CT* computed tomography, *DCI* delayed cerebral ischemia. *Fisher group* refers to the original Fisher scale, *XeCT* xenon-enhanced computed tomography

### Conditions During XeCT Procedures

Hemodynamic and respiratory parameters were clinically stable with small alterations from *start* to *end* in the XeCT procedures performed (*n* = 159). The use of vasoactive support was generally low; dobutamine was used at the time of measurement in 24 XeCT procedures (dose range 1–12 μg/kg/min) and norepinephrine (separately or combined) in 24 procedures (dose range 0.01–0.38 μg/kg/min). No adverse events were observed in relation to the procedures.

Comparison of the conditions *over time* at XeCT day 0–3 and 4–7 for the s*ubgroup 04* (*n* = 51) showed MAP 89.9 (CI 87.3–92.6) mmHg versus 94.3 (CI 90.9–97.7) (*P* = 0.014), mean *p*CO_2_ 5.20 (CI 5.08–5.33) kPa versus 5.60 (CI 5.43–5.76) (*P* < 0.001), and mean dose of propofol sedation 2.6 (CI 2.2–2.9) mg/kg/h versus 2.50 (CI 2.12–2.87).

When comparing the groups with *high or low initial CBF* at baseline (day 0–3), mean MAP was 89.9 (CI 86.0–93.9) mmHg versus 89.9 (CI 86.3–93.6), mean pCO_2_ 5.05 (CI 4.82–5.29) kPa versus 5.28 (CI 5.13–5.43), and mean dose of propofol sedation was 2.4 (CI 1.9–3.0) mg/kg/h versus 2.7 (CI 2.2–3.1) for the high- and low-CBF groups, respectively, and the differences were nonsignificant.

Details of the systemic physiological parameters, sedation dose, and neurological grade for the different groups and time windows compared are presented in conjunction with CBF measurements in Table 2 and 3 as described in the sections below.

**Table 2:** Calculated XeCT CBF parameters for patients with measurements at day 0–3 and day 4–7 (subgroup 04, *n* = 51)

Subgroup 04	High early CBF (*n* = 34)				Low early CBF (*n* = 17)			
	Day 0–3		Day 4–7		Day 0–3		Day 4–7	
	Median	(IQR)	Median	(IQR)	Median	(IQR)	Median	(IQR)
Glob CBF (ml/100 g/min)	37.7	(32.6–46.7)	36.8	(29.4–44.8)	23.6	(21.0–28.1)	28.4	(22.7–38.3)
					*P* = 0.025			
rCBF worst territory (ml/100 g/min)	27.7	(22.5–39.1)	28.9	(19.8–34.3)	16.2	(11.2–19.8)	20.0	(16.8–29.1)
					* P* = 0.017			
% ROI area (rCBF < 20 ml/100 g/min)	10.0	(2.8–17.3)	8.3	(1.2–30.4)	39.4	(25.7–50.3)	25.6	(8.9–41.0)
					*P* = 0.042			
% ROI area (rCBF < 10 ml/100 g/min)	0.0	(0.0–3.7)	0.0	(0.0–7.7)	7.4	(1.7–13.4)	3.5	(0.7–5.0)

	Mean	(CI)	Mean	(CI)	Mean	(CI)	Mean	(CI)
MAP (mmHg)	90.2	(84.5–96.0)	93.1	(89.4–96.8)	88.8	(83.0–94.5)	92.0	(85.2–98.8)
PaCO_2_ (mmHg)	39.4	(38.3–40.5)	41.5	(39.8–43.2)	38.0	(36.1–40.0)	42.4	(40.7–44.2)
ETCO2 (mmHg)	37.8	(36.4–39.2)	39.0	(37.6–40.4)	35.3	(33.1–37.4)	38.9	(36.3–41.6)
PaO2/FiO_2_ (mmHg)	262	(234–290)	255	(224–285)	247	(206–288)	243	(204–281)
Hematocrit (%)	34.6	(33.4–35.9)	33.2	(31.9–34.5)	34.7	(33.0–36.5)	33.2	(31.8–34.7)
Propofol dose (mg/kg/h)	2.2	(1.5–2.9)	2.3	(1.6–3.0)	2.7	(2.3–3.1)	2.1	(1.4–2.8)

	Patients	(%)	Patients	(%)	Patients	(%)	Patients	(%)
HH grade IV–V at time of XeCT	22	(65)	25	(74)	13	(76)	11	(65)
Clinical diagnosis of DCI	–		13	(38)	–		9	(53)

Patients are stratified by high or low early global CBF at day 0–3 (cutoff 30 ml/100 g/min). Systemic physiological parameters and sedation dose at the start of the XeCT procedures

*CBF* cerebral blood flow, *CI* confidence interval, *DCI* delayed cerebral ischemia, *ETCO*_2_ end-tidal CO_2_, *Glob* global, *HH grade* Hunt and Hess grade, *IQR* interquartile range, *MAP* mean arterial pressure, *PaCO*_*2*_ arterial PCO_2_, *PaO*_2_/*FiO*_2_ ratio arterial PO_2_/inspired fraction O_2_, *r* regional, *rCBF* regional cerebral blood flow, *ROI area* region-of-interest area, *XeCT* xenon-enhanced computed tomography

**Table 3:** Calculated XeCT-CBF parameters at the different phases of the acute course after SAH day 0–3 and 4–7 (subgroup 04)

Standard treatment	High early CBF (*n* = 21)				Low early CBF (*n* = 8)			
	Day 0–3		Day 4–7		Day 0–3		Day 4–7	
	Median	(IQR)	Median	(IQR)	Median	(IQR)	Median	(IQR)
Glob CBF, ml/100 g/min	40.0	(34.9–48.5)	35.0	(28.6–41.4)	27.1	(21.7–28.7)	26.6	(21.9–28.9)
	*P* = 0.002				n.s.			
rCBF worst territory (ml/100 g/min)	30.4	(22.9–33.0)	24.2	(18.9–30.3)	19.0	(15.7–22.5)	17.0	(16.3–19.6)
	*P* = 0.004							
% ROI area (rCBF < 20 ml/100 g/min)	10.0	(2.4–16.5)	10.0	(0.9–31.8)	25.7	(23.1–47.2)	30.4	(24.3–41.8)
% ROI area (rCBF < 10 ml/100 g/min)	1.3	(0.0–5.0)	0.0	(0.0–8.3)	4.2	(1.2–7.6)	4.4	(3.4–5.9)

	Mean	(CI)	Mean	(CI)	Mean	(CI)	Mean	(CI)
MAP (mmHg)	88.4	(84.0–92.8)	94.7	(89.0–100.3)	90.5	(84.6–96.3)	94.2	(87.2–101.3)
PaCO_2_ (mmHg)	38.7	(37.1–40.3)	43.0	(40.8–45.3)	37.6	(35.1–40.1)	41.7	(38.2–45.1)
Hematocrit (%)	34.6	(32.8–36.5)	33.9	(32.3–35.3)	33.3	(30.2–36.4)	33.1	(31.0–35.2)
Propofol dose (mg/kg/h)	2.2	(1.5–2.9)	2.3	(1.6–3.0)	2.1	(1.0–3.3)	1.8	(0.8–2.7)

Clinical DCI, HHH therapy	High early CBF (*n* = 13)				Low early CBF (*n* = 9)			
	Day 0–3		Day 4–7		Day 0–3		Day 4–7	
	Median	(IQR)	Median	(IQR)	Median	(IQR)	Median	(IQR)
Glob CBF ml/100 g/min	34.5	(31.9–41.7)	39.4	(33.9–48.8)	21.3	(20.8–25.9)	37.8	(23.6–41.0)
					*P* = 0.008			
rCBF worst territory ml/100 g/min	26.5	(22.2–33.0)	30.0	(21.1–40.7)	14.3	(9.9–17.3)	25.2	(19.4–29.9)
					*P* = 0.008			
% ROI area (rCBF < 20 ml/100 g/min)	11.3	(3.8–22.5)	3.3	(1.2–21.7)	46.7	(34.8–50.8)	17.8	(7.2–39.2)
					*P* = 0.021			
% ROI area (rCBF < 10 ml/100 g/min)	0.0	(0.0–1.4)	0.0	(0.0–2.1)	8.7	(4.7–21.7)	1.8	(0.0–4.9)

	Mean	(CI)	Mean	(CI)	Mean	(CI)	Mean	(CI)
MAP (mmHg)	93.4	(86.6–100.3)	93.0	(86.9–99.0)	89.4	(82.9–96.0)	95.2	(82.0–108.4)
PaCO_2_ (mmHg)	40.3	(38.7–41.9)	38.8	(36.9–40.8)	38.4	(34.9–41.9)	43.0	(40.7–45.4)
Hematocrit (%)	34.7	(32.8–36.5)	32.5	(29.9–35.1)	36.2	(34.7–37.7)	33.3	(30.9–35.7)
Propofol dose (mg/kg/h)	2.2	(1.5–2.9)	2.3	(1.6–3.0)	2.7	(2.3–3.1)	2.1	(1.4–2.8)

Patients are stratified by high or low early global CBF at day 0–3 (cutoff 30 ml/100 g/min) *and* by whether HHH therapy *was given* during the ICU course. Systemic physiological parameters and sedation dose at the start of the XeCT procedures

*CBF* cerebral blood flow, *CI* confidence interval, *DCI* delayed cerebral ischemia, *Glob* global, *IQR* interquartile range, *MAP* mean arterial pressure, *PaCO*_*2*_ arterial PCO_2_, *r* regional, *rCBF* regional cerebral blood flow, *ROI area* region-of-interest area, *XeCT* xenon-enhanced computed tomography

### CBF at Day 0–3, Baseline

**Fig. 2:**
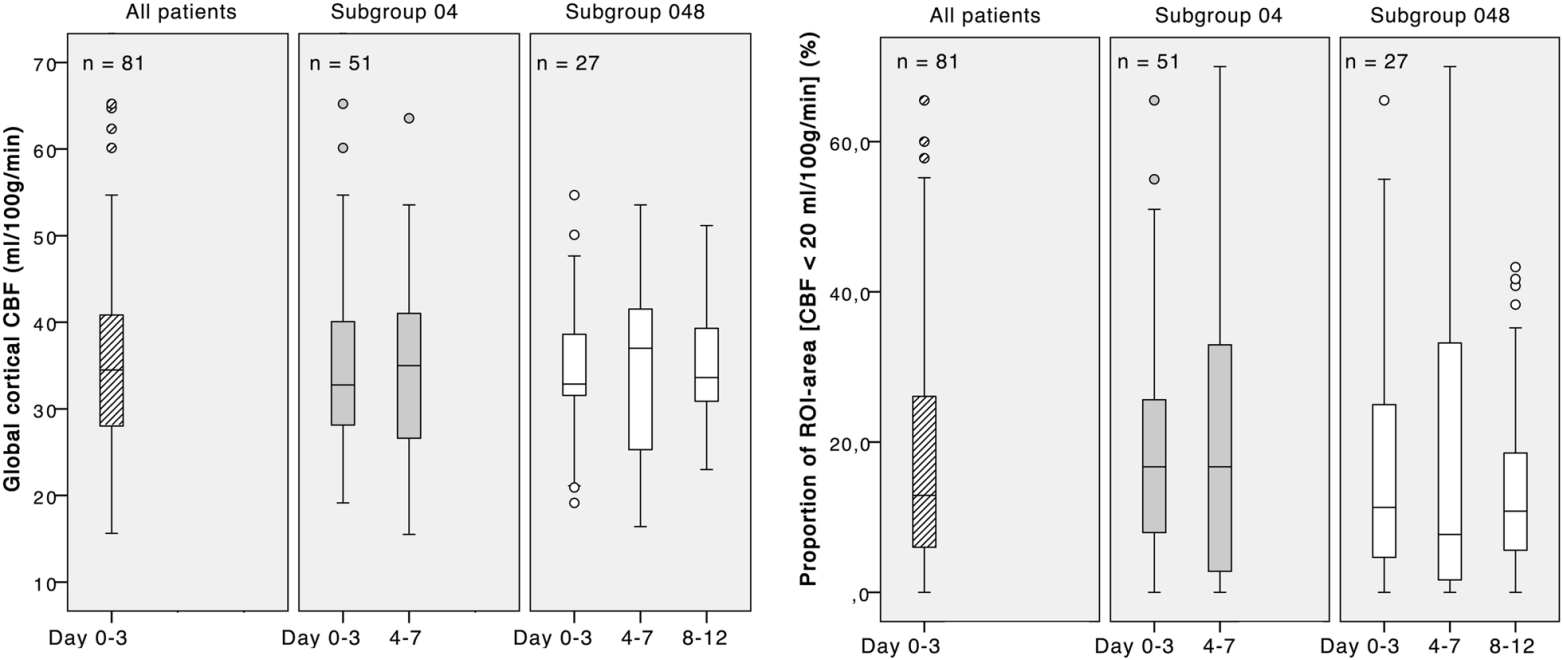
Global cortical CBF (left) and proportion of low-flow ROI area with local CBF < 20 ml/100 g/min (right) at different phases in the acute course of SAH. The separate panels in each graph show boxplots (median, IQR) for *all* patients and the *subgroups 04 and 048* with measurements at day 0–3, 4–7, and 8–12, respectively

As shown in Fig. 2, median global cortical CBF at day 0–3 for all patients (*n* = 81) was 34.5 (IQR 27.9–41.0) ml/100 g/min, and the median proportion of ROI area with local CBF below the threshold of 20 ml/100 g/min was 12.9% (IQR 5.5–28.3%).

### Course Over Time: CBF Parameters at Day 0–3 Versus 4–7 and 8–12

For the *subgroup 04* with measurements at day 0–3 *and* 4–7 (*n* = 51), median global cortical CBF was 32.8 (IQR 28.0–40.1) ml/100 g/min at day 0–3 versus 35.0 (IQR 25.4–41.3) at day 4–7. The proportion of low-flow ROI area was 16.7% (IQR 6.7–26.1) versus 16.7% (IQR 2.3–34.1) for day 0–3 and 4–7, respectively (Fig. 2).

For the *subgroup 048* with measurements at all three phases, day 0–3, 4–7 and 8–12 (*n* = 27), global cortical CBF was 32.9 (IQR 31.5–39.8) ml/100 g/min at day 0–3 versus 37.0 (IQR 25.2–41.7) day 4–7 versus 33.6 (IQR 30.8–39.6) ml/100 g/min at day 8–12. Low-flow ROI area at the different time intervals for this subgroup constituted 11.3% (IQR 3.3–25.0) versus 7.7% (IQR 1.6–34.7) and 10.8% (IQR 5.0–18.8), respectively (Fig. 2).

The differences in CBF parameters comparing measurements between the different time windows for patients in both subgroup 04 and subgroup 048 showed a wide range and did not reach statistical significance.

### CBF Course Depending on High or Low Early Baseline CBF

**Fig. 3:**
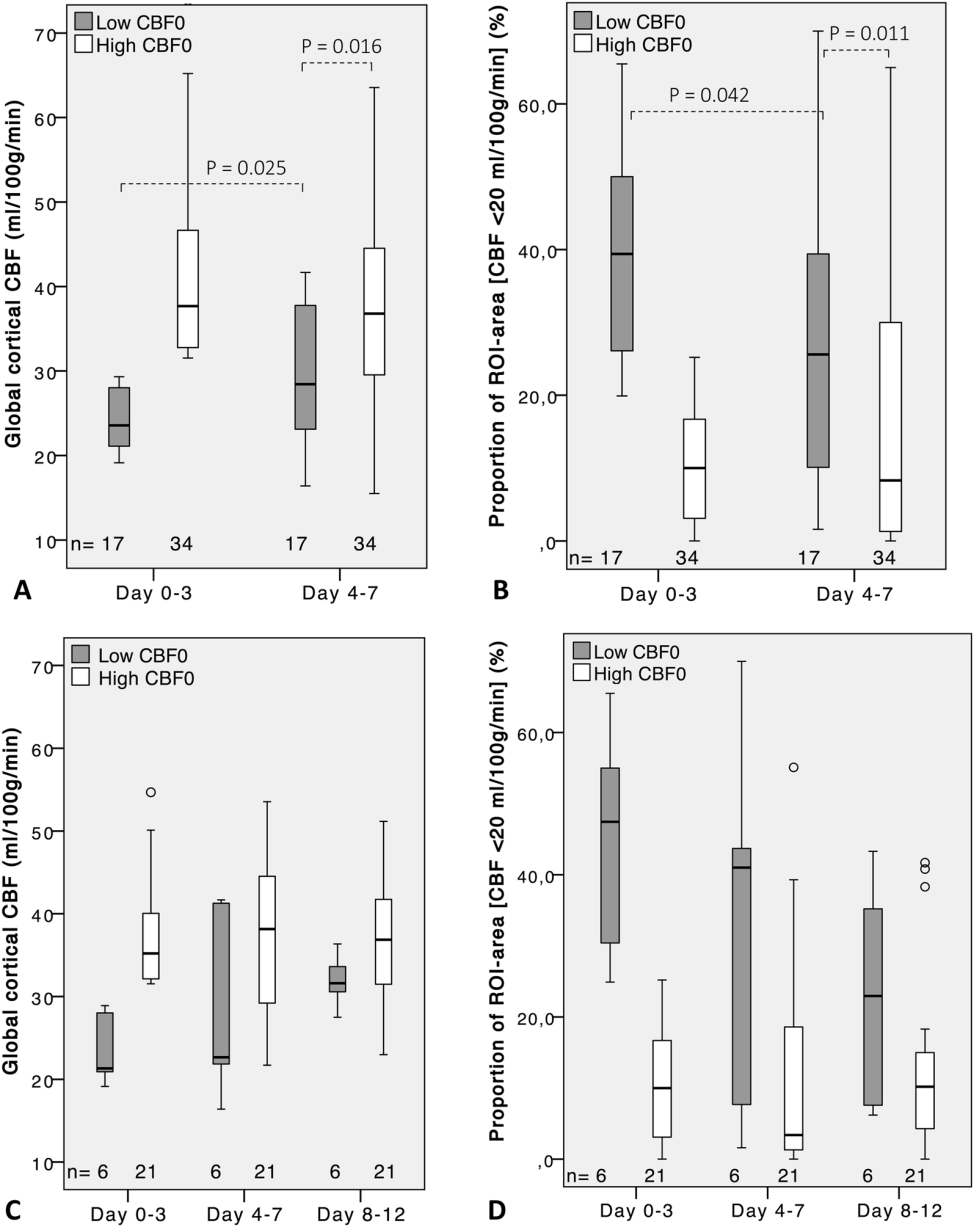
Global cortical CBF and proportion of ROI area with local CBF < 20 ml/100 g/min at different phases in the acute course of SAH for patients grouped by high or low early global CBF at day 0–3, cutoff 30 ml/100 g/min. **a**, **b** Boxplots (median, IQR) for *subgroup 04* (*n* = 51) with measurements at day 0–3 and 4–7. **c**, **d** Boxplots for *subgroup 048* (*n* = 27) with measurements at day 0–3, 4–7, and 8–12

Patients were dichotomized into a high- and a low-CBF group depending on the baseline global cortical CBF day 0–3, cutoff 30 ml/100 g/min. Among the 51 patients with two or more XeCT measurements, there were 17 patients with low initial CBF and 34 with high CBF. Global cortical CBF, regional CBF of the worst vascular territory, and the proportion of low-flow area for these groups are presented in Fig. 3a, b and Table 2.

*Day 0*–*3 versus day 4*–*7*—Median global cortical CBF for the high-CBF group was 37.7 (IQR 32.6–46.7) ml/100 g/min at day 0–3 compared to 36.8 (IQR 29.5–44.8) at day 4–7 (Fig. 3a, b). In the low-CBF group, CBF increased from 23.6 (IQR 21.0–28.1) ml/100 g/min to 28.4 (IQR 22.7–38.3) at day 4–7 (*P* = 0.025), still markedly lower compared to the high-CBF group (*P* = 0.016). The rCBF of worst vascular territory (Table 2) followed a similar pattern and was unchanged over time for the high-CBF group but increased from 16.2 (IQR 11.2–19.8) ml/100 g/min to 20.0 (IQR 16.8–29.1) at day 4–7 for the low-CBF group (*P* = 0.017). The proportion of ROI area with CBF below 20 ml/100 g/min also remained statistically unchanged for the high-CBF group, 10.0% (IQR 2.8–17.3) day 0–3 versus 8.3% (IQR 1.2–30.4) day 4–7. The low-CBF group showed a decrease in the proportion of low-flow ROI area from 39.4% (IQR 25.7–50.3) day 0–3 to 25.6% (IQR 8.9–41.0) day 4–7 (*P* = 0.042).

*Day 0*–*3 versus day 4*–*7 versus day 8*–*12*—In the subset of 27 patients who had measurements at all three time windows, six patients had low initial CBF. In the high-CBF group, global cortical CBF and proportion of ROI area with CBF below 20 ml/100 g/min were statistically unchanged through the three measurements; median CBF 35.2 (IQR 32.0–41.8) ml/100 g/min versus 38.1 (IQR 28.6–45.0) versus 36.9 (IQR 31.1–42.2) (Fig. 3c, d). In the low-CBF group in this subset of patients, median CBF was 21.3 (IQR 20.5–28.2) ml/100 g/min versus 22.7 (20.5–28.2) versus 31.6 (IQR 29.8–34.4), but the increase did not reach statistical significance when analyzed with time window as independent variable. A corresponding numeric decrease in proportion of low-flow ROI area for this group from day 0–3 to day 8–12 was also found statistically nonsignificant (Fig. 3d).

### CBF Course Depending on Whether HHH Therapy was Given (Day 0–3 vs. Day 4–7)

Twenty-two of the 51 patients with repeated XeCT procedures were receiving HHH therapy due to clinical suspicion of DCI. As the clinical course and CBF dynamics may differ from the natural course of patients with “standard treatment”, patients were further divided into subgroups according to whether HHH therapy was given or not. The more detailed CBF data and physiological parameters for these subgroups are presented in Table 3.

#### Standard Treatment

Among the patients (*n* = 21) with high initial CBF and standard treatment (i.e., *no* clinical suspicion of DCI), global cortical CBF was 40.0 (IQR 34.9–48.5) ml/100 g/min at day 0–3 versus 35.0 (IQR 28.6–41.4) ml/100 g/min at day 4–7 (*P* = 0.002). In the group with low initial CBF and standard treatment (*n* = 8), the initial CBF was 27.1 (IQR 21.7–28.7) ml/100 g/min versus 26.6 (IQR 21.9–28.9) at day 4–7 (Table 3).

#### HHH Therapy

Patients with high initial CBF, who subsequently received HHH therapy (*n* = 13), had a slight, but nonsignificant, increase in median CBF from 34.5 (IQR 31.9–41.7) ml/100 g/min day 0–3 to 39.4 (IQR 33.9–48.8) day 4–7. In the group with low initial CBF and subsequent HHH therapy (*n* = 9), CBF increased markedly from 21.3 (IQR 20.8–25.9) ml/100 g/min day 0–3 to 37.8 (IQR 23.6–41.0) day 4–7 (*P* = 0.006) (Table 3).

Regional CBF parameters followed a similar pattern, as seen in Table 3, where the most pronounced change from day 0–3 to 4–7 was in patients with low initial CBF who received HHH therapy; the median proportion of ROI area with CBF below 20 ml/100 g/min was reduced from 46.7% (IQR 34.8–50.8) to 17.8% (IQR 7.2–39.2) (*P* = 0.021). In patients with low initial CBF and standard treatment, the proportion of low-flow ROI area was statistically unchanged; median 25.7% (IQR 23.1–47.2) day 0–3 versus 30.4% (IQR 24.3–41.8) day 4–7. For patients with high initial CBF, the proportion of low-flow ROI area remained at a lower level in both the standard treatment and the HHH therapy groups.

### Early Clinical Course Outcome

In the group of patients with low initial CBF (< 30 ml/100 g/min) at day 0–3, thirteen of 29 patients (45%) had poor clinical course outcome at discharge from NIC (unconscious, GCS motor ≤ 5, or dead) compared to 18 of the 52 patients (35%) with high initial CBF. In the subgroup 04 with repeated XeCT procedures, poor outcome was concluded in 11 of the 20 patients (55%) who still had low CBF at XeCT day 4–7 compared to 11 of 31 patients (35%) with high CBF at day 4–7. The differences in proportion of patients with poor outcome between the low- and high-CBF groups did not reach statistical significance.

Radiologically, the proportion of patients with infarcts with a largest diameter exceeding 20 mm at follow-up CT was 34% in the group with low baseline CBF compared to 44% in the high-CBF group. For the subgroup 04, when CBF at day 4–7 was considered, the occurrence of infarcts > 20 mm was 60% in low-CBF patients and 35% in high-CBF patients. Neither of these differences reached statistical significance.

## Discussion

Impairment of CBF following SAH has been demonstrated in several experimental and clinical studies [20–23]. In an early study on the course of CBF over time after SAH by Meyer et al. [24], CBF was typically found diminished immediately after the hemorrhage and then declined further to reach its lowest level at approximately day 7–10, followed by a slow restitution of CBF over several weeks. The referenced study included predominantly good-grade patients, who were not intubated. Several clinical studies of CBF have been focused on the early acute phase after SAH, evaluating the possibility to predict the occurrence of DCI or outcome [25, 26], and yet other studies were aimed to evaluate the effect of interventions [27]. The aim of our present study was to assess the temporal dynamics of global and regional CBF in *poor*-*grade*, mechanically ventilated SAH patients during their course in NIC, while also considering possible different pathways depending on the initial level of CBF and influence from HHH therapy given on suspicion of DCI.

The baseline cortical CBF (median 34.5 ml/100 g/min for all patients) was in accordance with previous studies for moderately sedated poor-grade SAH patients [21, 26]. The changes in CBF parameters over time during the acute course of SAH were small when comparing data for *all* patients. Hence, we did not find the hypothesized pattern with a decrease in CBF at day 4–7. This may correspond to the findings in another early study by Jakobsen et al. [20] that CBF of the most severely graded SAH patients is already initially very low with a slow recovery during the acute course in contrast to the pattern of good-grade patients. It appears important not only to study temporal CBF dynamics in SAH patients all together but also specifically in different subgroups depending on severity grade and clinical management.

When patients were dichotomized into a low- and high-CBF group according to their baseline measurements, patients with high initial CBF had no significant changes in CBF parameters from baseline to day 4–7. However, the low-CBF group showed a moderate increase in CBF at day 4–7, but to a level still significantly lower compared to the high-CBF group. There were also an increase in rCBF of worst vascular territory and a corresponding decrease in low-flow ROI area at day 4–7 for the low-CBF group. In the subset of patients also examined at day 8–12, there was an apparent further increase in median CBF for the low-CBF group, but the number of patients was small, and the change was not statistically significant. These findings may be of clinical importance since the identified subgroups may require different surveillance and treatment.

It is likely important to consider the effects of instituted therapy when CBF dynamics is studied. A significant number of patients received HHH therapy on clinical suspicion of DCI. Even though the actual effects of HHH therapy are still controversial [28], it is likely that this intervention may influence regional and global CBF in some patients. Among patients who received *HHH therapy* during the course, there was no significant change in CBF for patients with high initial CBF, but a marked increase for patients with low initial CBF. A corresponding pattern was found for the proportion of low-flow ROI area for the respective groups. As this study was not designed to evaluate HHH therapy, these results should not be regarded as proof of its effects, but rather an observed possible influence of HHH therapy on CBF in patients with initially low CBF. It cannot be ruled out that these patients were initially hypovolemic or had a natural recovery of CBF after the early impact. For the subset of patients with XeCT at all three time windows, further division in subgroups unfortunately rendered too few patients in each group for meaningful analysis of this aspect.

Among patients with *standard treatment*, those with high initial CBF showed a moderate decrease in CBF at day 4–7, whereas patients with low initial CBF remained at low CBF at day 4–7. It is plausible that DCI may be underdiagnosed in intubated, unconscious patients and that some of the standard treated patients with persistent low CBF actually were suffering from unrecognized DCI.

As this is a small study, we did not intend to specifically study outcome related to CBF parameters. However, to reflect the short-term outcome of the different groups, neurological state at discharge from NIC was determined. The numeric difference found in the proportion of patients with poor outcome between the high- and low-CBF groups did not reach statistical significance but the picture was that a larger proportion of patients with low CBF at day 0–3 and day 4–7 had poor outcome. The occurrence of infarcts on follow-up CT was numerically higher in patients with high baseline CBF, but when the CBF at day 4–7 was considered, the occurrence was higher in low-CBF patients. The differences did not reach statistical significance but may indicate that development of infarcts is more dependent on the CBF level later in the course.

### Limitations

Despite the intention to consecutively include all intubated SAH patients, there is always a risk of selection bias. The main reasons for not including patients were logistic factors and medical priorities, so it is unlikely that the selection of patients influenced the results in an improper way. It should, however, be stressed that only poor-grade SAH patients in need of mechanical ventilation were studied, and the results are not generalizable to the entire population of SAH patients.

We used data from patients who had valid XeCT procedures at one, two, or three consecutive time windows. From the clinical characteristics (Table 1), it is noted that the proportion of patients with higher severity grade is slightly larger in the subset of patients (*n* = 27) who were examined three times. This is expected, as only patients still in need of ventilation day 8–12 had a third XeCT, and data for this group may thus represent the patients with most severe SAH. Accordingly, the study lacks information about CBF dynamics in patients who improved sufficiently to be extubated before the second or third time window.

There were generally small differences in systemic physiological conditions between the groups and time windows studied. For the subgroups with measurements also at day 4–7 and 8–12, there were statistically significant differences in mean MAP and pCO_2_ between the time windows. The differences were modest from a clinical view, but influence on CBF from these factors is possible. There were, however, no significant differences in these parameters between the groups defined by high or low initial CBF.

## Concluding Remarks

Global and regional cortical CBF studied in poor-grade SAH patients *at large* did not show any statistically significant changes over time during the acute course of SAH. When patients were stratified according to high or low initial CBF (at day 0–3), a pattern was revealed where low initial CBF was associated with a persistent low level of CBF at day 4–7. The association was more pronounced when patients with standard treatment were separated from those receiving HHH therapy. These findings may be of clinical relevance for poor-grade SAH patients found to have low early CBF, who could benefit from careful surveillance and optimization of circulation. The findings also illustrate the importance of breaking down the analysis into subgroups since the overall results may not be representative for all included patients.
